# Neuroprotective Effects of Diabetes Drugs for the Treatment of Neonatal Hypoxia-Ischemia Encephalopathy

**DOI:** 10.3389/fncel.2020.00112

**Published:** 2020-05-06

**Authors:** Laura Poupon-Bejuit, Eridan Rocha-Ferreira, Claire Thornton, Henrik Hagberg, Ahad A. Rahim

**Affiliations:** ^1^UCL School of Pharmacy, University College London, London, United Kingdom; ^2^Centre for Perinatal Medicine and Health, Institute of Clinical Sciences, Sahlgrenska Academy, University of Gothenburg, Gothenburg, Sweden; ^3^Department of Comparative Biomedical Sciences, Royal Veterinary College, London, United Kingdom

**Keywords:** hypoxic-ischemic encephalopathy, perinatal brain injury, cerebral palsy, neuroprotection, hypothermia, diabetes

## Abstract

The perinatal period represents a time of great vulnerability for the developing brain. A variety of injuries can result in death or devastating injury causing profound neurocognitive deficits. Hypoxic-ischemic neonatal encephalopathy (HIE) remains the leading cause of brain injury in term infants during the perinatal period with limited options available to aid in recovery. It can result in long-term devastating consequences with neurologic complications varying from mild behavioral deficits to severe seizure, intellectual disability, and/or cerebral palsy in the newborn. Despite medical advances, the only viable option is therapeutic hypothermia which is classified as the gold standard but is not used, or may not be as effective in preterm cases, infection-associated cases or low resource settings. Therefore, alternatives or adjunct therapies are urgently needed. Ongoing research continues to advance our understanding of the mechanisms contributing to perinatal brain injury and identify new targets and treatments. Drugs used for the treatment of patients with type 2 diabetes mellitus (T2DM) have demonstrated neuroprotective properties and therapeutic efficacy from neurological sequelae following HIE insults in preclinical models, both alone, or in combination with induced hypothermia. In this short review, we have focused on recent findings on the use of diabetes drugs that provide a neuroprotective effect using *in vitro* and *in vivo* models of HIE that could be considered for clinical translation as a promising treatment.

## Introduction

Hypoxic-ischemic encephalopathy (HIE) is the most common neonatal encephalopathy accounting for up to 85% of cases (Volpe, 2012). It is caused by of an inadequate oxygen supply and blood flow resulting in a variety of clinical manifestations (Ferriero, 2004; Allen and Brandon, 2011; Hagberg et al., 2015). These include developmental delays, epilepsy, cerebral palsy, and death (Dilenge et al., 2001; Shankaran, 2012; Hagberg et al., 2016). One to six babies per 1,000 live births in high-income countries and approximately 20 infants per 1,000 live births in low- and middle-income countries die or develop a life-long brain condition. This accounts for approximately one million deaths annually (Lee et al., 2013; Pauliah et al., 2013; Wu et al., 2014).

Currently, the standard care for neonates with HIE is therapeutic hypothermia (TH), which is able to reduce overall neurodevelopmental disability and mortality (Jacobs et al., 2013; Azzopardi et al., 2014; Silveira and Procianoy, 2015; Rao et al., 2017). However, while TH is very promising, up to 55% of treated neonates are not protected and still develop life-long neurodisabilities, including cerebral palsy (Jacobs et al., 2013; Davidson et al., 2015). Therefore, there is a need to develop therapies that are either more effective than hypothermia, can be used in combination with hypothermia to enhance its therapeutic efficacy, or which can be used alone in lower resource environments.

**FIGURE 1:**
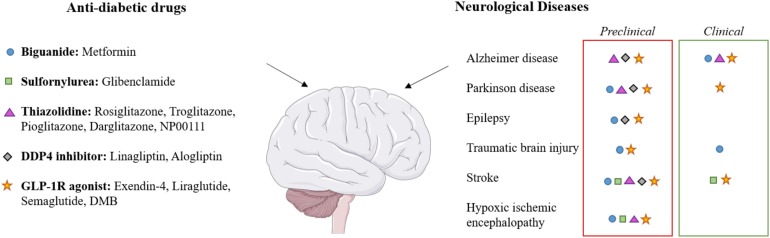
Effects of diabetes drugs on various neurological diseases. The applied color code indicates whether the effect on the target disease has been observed in preclinical studies (red boxes) or in clinical studies (green boxes). Each class of drugs corresponds to a symbol which is indicated in neurological diseases for which clinical and/or preclinical studies has been performed. Brain image taken from the SMART Servier Medical Art Library (https://smart.servier.com).

Over the past decade, a growing number of pre-clinical and now clinical studies have provided evidence of drugs licensed for the treatment of diabetes as having protective effects on the brain (Athauda et al., 2017; Rotermund et al., 2018; Mousa and Ayoub, 2019). These effects have been proven in different neurological conditions such as Alzheimer’s disease (AD) and Parkinson’s Disease (PD), traumatic brain injury (TBI), stroke and epilepsy (Figure 1). Given the need to develop effective treatments for neonatal HIE, researchers have investigated these diabetes drugs to assess their therapeutic efficacy for this indication.

In this short review, we first describe the experimental and animal models of HIE that are used in preclinical studies to assess the therapeutic efficacy of candidate drugs (Table 1). We then highlight the studies that support the potential of commonly used diabetes medicines to ameliorate neurological damage from HIE. This includes recent data demonstrating that diabetes drugs can enhance the therapeutic effect of TH.

**TABLE 1:** Evidence supporting the neuroprotective properties of diabetes drugs use in the context of treatments for hypoxia-ischemia encephalopathy.

**Diabetes drug**	**Model**	**Species**	**Treatment**	**Effects**	**References**
Biguanide	OGD	bEND.3 cells	Metformin	Inhibition of inflammatory signaling pathways	Liu et al., 2014
Biguanide	OGD	Rat cortical neurons	Metformin	Pre-treatment of neurons alleviated OGD/R-induced injury	Meng et al., 2016
Biguanide	OGD	Primary rat fetal-derived astrocytes	Metformin	Improve cell viability via reduction of apoptosis mechanisms	Gabryel and Liber, 2018
Biguanide	OGD	PC-12 cells	Metformin	Reduce cell death under OGD/R condition and attenuation of ROS generation	Mohammad Alizadeh et al., 2018
Biguanide	OGD	Primary cortical and hippocampal neurons	Metformin	Moderate improvement of cell viability	Mielke et al., 2006
TZD	OGD	Rat hippocampal slices	NP00111 Rosiglitazone	Protection against OGD by a mechanism related to phosphorylation of ERK1/2 via activation of PPARγ	Rosa et al., 2008
TZD	OGD	Primary cultured astrocytes	Pioglitazone	Protective effects with inhibition of pyroptosis mechanism induced by the OGD	Xia et al., 2018
DPP4	OGD	HBMVECs	Alogliptin	Protection against OGD and increasing of permeability in human brain vascular cells	Hao et al., 2019
Incretin GLP1-R agonist	OGD	Rat cortical neurons	Exendin-4	Protects neurons through PKA pathway	Wang M.-D. et al., 2012
Incretin GLP1-R agonist	OGD	Rat cortical neurons	Liraglutide	Neuroprotective action with reduction of apoptosis and ROS via activation of the PI3K/AKT and MAPK pathways	Zhu et al., 2016
Incretin GLP1-R agonist	OGD	Mouse cortical neurons	DMB	Neuroprotection with anti-apoptotic effects, mediated by activation of the GLP-1R through the cAMP-PKA-CREB signaling pathway	Zhang et al., 2016
Sulfonylurea	HI P10	Rat	Glibenclamide	No effects on severe HI model Improvement neurological functions in moderate HI model	Zhou et al., 2009
TZD	HI 8W	Ob/Ob mouse	Darglitazone	Reduction of the infarct size and neuroinflammation response	Kumari et al., 2010
Biguanide	HI P8	Mouse	Metformin	Activation of endogenous NPCs, promoting their migration and differentiation in the injured brain Restoration of sensory-motor function	Dadwal et al., 2015
Biguanide	HI P3	Rat	Metformin	Attenuation of cognitive impairments Induction of OPCs proliferation reducing myelination damage	Qi et al., 2017
Biguanide	HI P7	Rat	Metformin	Attenuation brain infarct and oedema Inhibition of neuronal apoptosis, and neuroinflammation + amelioration of the blood brain barrier breakdown	Fang et al., 2017
Biguanide	HI P8	Mouse	Metformin	Sex-dependent effects on proliferation but increases neurogenesis in both sexes; rescues cognitive deficits in adult females	Ruddy et al., 2019
Incretin GLP1-R agonist	HI P7/P10	Mouse	Exendin-4**	Neuroprotective effect alone or in combination with therapeutic hypothermia	Rocha-Ferreira et al., 2018
Incretin GLP1-R agonist	HI P7	Rat	Liraglutide	Inhibited apoptosis and promoted neuronal survival; PI3K/Akt pathway involved	Zeng et al., 2020

In vitro model with OGD on different cell types and *in vivo* models with HI at different stage of age. **Only study using combination with hypothermia.

## Experimental and Animal Models of HIE

Hypoxic-ischemic encephalopathy is an evolving process that involves distinct phases leading to a delayed cell death, including primary injury, latent phase, secondary phase, and tertiary phase (Wyatt et al., 1989; Fleiss and Gressens, 2012; Davidson et al., 2015). Understanding the characteristics observed during the different phases leading to neonatal encephalopathy are key to the development of new therapeutics, when they can be used to ameliorate HIE and the multiple possible subsequent sequela. The timing of the events following hypoxia-ischemia (HI) and the therapeutic window in rodent models is well defined at ∼6 h correlating with initiation of the secondary phase of brain injury (Nair and Kumar, 2018). Therefore, there is a narrow window within the first few hours of birth during which a therapy should be initiated for optimal outcomes (Silveira and Procianoy, 2015; Martinello et al., 2017). Furthermore, if the drug is administered systemically, then it should be able to reach the brain quickly and cross the blood brain barrier (BBB).

The neuroprotective properties of diabetes drugs were first recognized by positive neurological effects in type 2 diabetes mellitus (T2DM) patients under treatment (Grant et al., 2011) and now in various studies for the treatment of different neurological conditions (Hussien et al., 2018; Rotermund et al., 2018; Erbil et al., 2019). A number of studies have demonstrated that diabetes drugs are indeed capable of entering the brain following systemic administrations and mediating a physiological response, e.g., metformin (Lv et al., 2012), sulfonylurea (SUR) (Simard et al., 2012), thiazolidine (Grommes et al., 2013), dipeptidyl peptidase-4 (DPP-4) inhibitors (Mousa and Ayoub, 2019), and glucagon-like peptide-1 receptor (GLP1-R) agonists (Hunter and Hölscher, 2012).

Accurate and reliable *in vitro* and *in vivo* models of HIE are of utmost importance in determining the mechanisms of damage and also evaluating the efficacy of potential treatments. The development of a variety of *in vitro* and *in vivo* models of HIE have facilitated this process.

### Oxygen Glucose Deprivation

Oxygen glucose deprivation is widely used as a relatively convenient *in vitro* model for ischemia, stroke or HIE, showing similarities with the *in vivo* models of brain ischemia (Tasca et al., 2015). This primary neural cell or immortalized cell culture model has been used extensively to examine the cellular mechanisms mediating ischemia–reperfusion injury (Rousset et al., 2015; Gao et al., 2019). The OGD model is a simple process that firstly involves changes to the cell culture medium to exclude glucose. The cells are incubated in a hypoxic incubator with decreased O_2_ and increased N_2_ levels with a saturated humidity atmosphere at 37°C over a specific period of time. Thus, cultured cells subjected to hypoxia, fuel deprivation and then reoxygenation mimic the scenario of ischemia–reperfusion.

### Hypoxia-Ischemia Surgery

The rodent model of neonatal HIE was first validated by Rice et al. (1981) and has since been extensively used to identify mechanisms of brain injury resulting from perinatal HI (Vannucci and Vannucci, 2005). It is also used to test potential therapeutic interventions. The HIE model is a two-step process and involves the ligation of one common carotid artery followed by exposure to a hypoxic environment before restoration to normal atmospheric conditions. Traditional models of HIE have utilized rodents at postnatal day 7–10 as being roughly equivalent to a near-term or term human infant based on electrophysiological, neurochemical, cardiovascular, and metabolic criteria of brain development (Hagberg et al., 1997; Semple et al., 2013). There are a wide variety of HI animal models used to investigate different aspects of HIE. Examples of this include; rodents (Recker et al., 2009), rabbits (Derrick et al., 2004), term piglet (Rocha-Ferreira et al., 2016, 2017), preterm sheep (Nitsos et al., 2014), and non-human primates (Juul et al., 2007). To date, existing preclinical data using diabetes drugs as a treatment for HIE have only been performed on rodent models. Furthermore, rodents have limitations in simulating the range, accuracy, and physiology of clinical HI and the relevant systems neuropathology that contribute to the human brain injury pattern. Large animal models of perinatal HI can better replicate the conditions of human HIE (Koehler et al., 2018).Therefore, the availability of these larger animal models of HIE are an invaluable tool to evaluate the therapeutic efficacy of these candidate diabetes drugs prior to any clinical trials.

## Diabetes Drugs and Neurological Diseases

Pharmacologic therapy of T2DM has changed dramatically in the last 10 years, with new drugs and drug classes becoming available. Among the different categories of therapies for T2DM, metformin serves as the first line drug whereas other hypoglycaemic agents (SUR, thiazolidine, DPP4 inhibitor, incretin) are used as second line therapies, or in combination with metformin. Over the past three decades, numerous epidemiological studies have shown a clear association between T2DM and an increased risk of developing neurological disorders (NDs) such as AD (Li et al., 2015), PD (Khan et al., 2014), epilepsy/seizure (Yun and Xuefeng, 2013), and stroke (Putaala et al., 2011).

Various studies suggest a comorbid association between NDs and T2DM indicating that there could be shared underlying pathophysiological mechanisms. Using comparative analysis, several putative “shared pathways” have been indentified and demonstrated how the insulin signaling pathway is related to other significant ND pathways. These include the signaling pathways for neurotrophin (Tong et al., 2009; Karki et al., 2017), PI3K/AKT (Gabbouj et al., 2019), mTOR (Bryan and Bowman, 2017; Sun et al., 2019), and mitogen-activated protein kinase (MAPK) (Santiago and Potashkin, 2013; Karki et al., 2017) and how these pathways cross-talk with each other. Consequently, studies started to investigate T2DM treatments as neuroprotective strategies for different types of ND, including perinatal HIE.

### Metformin

Metformin is a biguanide drug widely used since the 1960s for the treatment of patients with T2DM. It enhances insulin sensitivity, induces glycolysis, and suppresses gluconeogenesis in the liver (Martin-Montalvo et al., 2013). Pre-clinical studies have also reported the promising therapeutic effect of metformin against neurodegeneration in conditions such as PD (Patil et al., 2014), epilepsy (Yimer et al., 2019), and cerebral ischaemia/reperfusion injury (Ge et al., 2017; Leech et al., 2019). In addition to its neuroprotective effects, metformin has been shown to promote neurogenesis by enhancing neural precursor self-renewal, proliferation, and differentiation (Potts and Lim, 2012; Wang J. et al., 2012). Increased neurogenesis upon metformin treatment resulted in improved memory formation in multiple experimental models of brain injury (Jin et al., 2014; Liu et al., 2014; Dadwal et al., 2015; Ge et al., 2017; Qi et al., 2017). *In vitro* studies using the OGD model demonstrated that metformin improves neuronal viability and regulates programmed cell death in a caspase-independent manner, thereby reducing ischemic reperfusion injuries (Mielke et al., 2006; Meng et al., 2016; Gabryel and Liber, 2018; Mohammad Alizadeh et al., 2018). Metformin treatment remarkably attenuated brain infarct volumes, brain oedema and restored behavior deficits in a neonatal rat model of HI (Qi et al., 2017). It also induced activation of endogenous neural precursor cells (NPCs) (Dadwal et al., 2015). A rodent model of neonatal HI injury showed better neuroprotection induced by metformin in females following early injury relative to males. Indeed, metformin treatment in mice increased the NPC pool in both sexes in neonates but only in females at the adult stage. Consequently, long-term metformin treatment leads to cognitive improvements in females, but not males following early HI injury (Ruddy et al., 2019). The mechanism could be linked to the sex hormones (Ruddy et al., 2019) but further exploration of the mechanism underlying this effect is required. Of note, females have an advantage following neonatal hypoxia ischemia; larger cognitive deficits and less functional recovery have been observed in males despite a comparable neuropathology across sexes (Smith et al., 2014). The neuroprotective properties of metformin were associated with inhibition of neuronal apoptosis, suppression of neuroinflammation and amelioration of the blood-brain barrier breakdown via downregulation of the NFκB signaling pathway (Fang et al., 2017). Overall, these studies have highlighted this drug as a promising potential treatment in childhood brain injury models.

### Sulfonylurea

Sulfonylurea agents are the second oral hypoglycaemic drugs after metformin and they remain an imperative tool for glucose control (Thulé and Umpierrez, 2014). Recent studies demonstrated that sulfonylurea receptor 1 (SUR1) is involved in brain injury in rodent models of stroke (Hussien et al., 2018). The SUR drugs glibenclamide and glimepiride have neuroprotective effects (Ortega et al., 2013; Wang et al., 2019) and ameliorate cerebral stroke, spinal cord injury, premature encephalopathy, and TBI (Tosun et al., 2013). The neuroprotective effect of SUR agents is not fully understood but glibenclamide blocks SUR1, a regulatory subunit of the microglial K_*ATP*_ channel. This channel is overexpressed in rodent models of stroke and the effect of blocking SUR1 could inhibit microglia activation which release inflammatory cytokines and initiate downstream signaling pathways, resulting in neuronal cell loss and necrosis (Ortega et al., 2013). Glibenclamide is clinically effective in preventing oedema and improving outcome after focal ischemia (Sheth et al., 2014); clinical studies are being conducted to evaluate its efficacy in acute cerebral embolism and severe cerebral edema (NCT03284463, NCT02864953). In a rat model of HI injury, glibenclamide improved several neurological parameters but failed to attenuate brain edema, infarct volume or brain tissue loss (Zhou et al., 2009). This may be attributed to the significant reduction in blood glucose induced by the dose of glibenclamide used, which may exacerbate the ischemic brain injury. More investigations need to be performed around the dose and its full potential as a treatment for childhood brain injury models.

### Thiazolidine

Thiazolidinedione [also called glitazone or peroxisome proliferator activated receptor-γ (PPARγ) agonists] are a group of oral anti-diabetic drugs designed to treat patients with T2DM. They enhance insulin sensitivity and reduce serum glucose in diabetic patients, without significant alterations in serum glucose of non-diabetic animals or humans (Plutzky, 2003). Rosiglitazone, troglitazone, and pioglitazone suppressed the activation and infiltration of macrophages and reduced the infarct size after cerebral ischemia in a middle cerebral artery occlusion (MCAO) model by reducing levels of proinflammatory cytokines (Sundararajan et al., 2005; Culman et al., 2007; Xia et al., 2018). Moreover, PPARγ agonists such as NP00111, rosiglitazone and pioglitazone treatments could relieve OGD-induced hypoxia injury *in vitro* and exert neuroprotective effects (Rosa et al., 2008; Xia et al., 2018). The neuroprotective effect of TZD requires further investigation. However, data suggests activation of PPARγ mediates suppression of NF-κB signaling pathway, inhibiting apoptosis and reducing neuronal loss (Zhang et al., 2011). In the context of adult HI injury, TZD has shown therapeutic efficacy in Ob/Ob mice, a model for T2DM and obesity. This model was chosen for its high risk factor of stroke and increased risk of brain damage. Darglitazone treatment in this adult diabetic mouse resulted in significant neuroprotection associated with a complete restoration of the initial microglial response and reduction of the infarct brain size at 24 h of recovery (Kumari et al., 2010). No studies have yet been conducted in a neonatal HI model but the proven neuroprotective properties and potent anti-ischemic effects of this class of diabetes drug could be a promising option.

### Incretin / GLP1-Receptor Agonists

Glucagon-like peptide-1-receptor agonists are used in combination with diet and exercise in the therapy of T2DM, either alone or in combination with other antidiabetic agents. GLP1-R agonists have been found to enter the brain following systemic administration (Hunter and Hölscher, 2012; Athauda et al., 2017) and have neuroprotective properties when assessed in various rodent models of neurological disease and damage such as AD (Xu et al., 2015; Cai et al., 2018), PD (Yun et al., 2018), epilepsy (Wen et al., 2019), TBI (Glotfelty et al., 2019), and stroke. As a result, a number of clinical trials are already underway for some of these molecules such as exendin-4 (NCT03456687, NCT02829502, NCT03287076), liraglutide (NCT02953665, NCT01469351, NCT01843075, NCT03948347) or semaglutide (NCT03659682) to assess benefits to AD, PD, or stroke patients. A PD trial is already completed and has reported that patients on exendin-4 show a statistically significant improvement in clinical motor and cognitive measures compared to the control group (Athauda et al., 2017). Numerous experimental studies also demonstrated the potential of glucagon-like peptide-1 (GLP1) and analog, such as liraglutide or semaglutide, to reduce acute ischaemic damage in the brain (Wang M.-D. et al., 2012; Zhu et al., 2016; Basalay et al., 2019; Yang et al., 2019). Exendin-4, liraglutide and quinoxaline 6,7-dichloro-2-methylsulfonyl-3-N-tert-butylaminoquinoxaline (DMB, an agonist and allosteric modulator of the GLP-1R) have been shown to increase neuron survival under OGD *in vitro* by reducing reactive oxygen species (ROS), apoptotic and necrotic mechanisms (Wang M.-D. et al., 2012; Zhang et al., 2016; Zhu et al., 2016). The potential that GLP1-R agonists have for treating perinatal HIE has been further strengthened in two recent studies demonstrating: (1) that exendin-4 has significant therapeutic efficacy in the mouse model of neonatal HIE (Rocha-Ferreira et al., 2018), (2) and that liraglutide exerts neuroprotection via the PI3k/Akt pathway (Zeng et al., 2020). The study by Rocha-Ferreira and colleagues demonstrated that systemic administration either directly after HI injury, or even 2 h later significantly reduced the size of the brain infarct, the inflammatory response and the oxidative stress. Exendin-4 treatment was able to work synergistically with hypothermia to further enhance therapeutic efficacy (Rocha-Ferreira et al., 2018).

### DPP-4 Inhibitor

Also called gliptins, DPP-4 inhibitors are a class of glucose-lowering agents for the treatment of T2DM. Their actions are mediated indirectly through preservation of GLP-1 incretins that are mainly metabolized by the key enzyme DPP-4 (Andersen et al., 2018). Preclinical studies have shown that DPP-4 inhibitors have neuroprotective effects (Darsalia et al., 2019; Mousa and Ayoub, 2019) but unlike GLP-1 receptor agonists (incretins), the ability of DPP-4 inhibitors to cross the blood-brain barrier are still unclear (Chen et al., 2015). However, they could indirectly increase levels of active GLP-1 in the brain that crosses from the blood (Yang et al., 2013). Several kinds of gliptins have been shown to be effective in different experimental models of neurological diseases such as AD (Wiciński et al., 2018; Dong et al., 2019), PD, epilepsy and stroke (Darsalia et al., 2019; Mousa and Ayoub, 2019). In the MCAO model mimicking stroke, linagliptin and alogliptin reduced infarct volume and neurological deficits (Chiazza et al., 2018; Hao et al., 2019). In the same study, alogliptin protectsed against oxygen glucose deprivation reperfusion (OGD/R) and has a neurovascular protective effect increasing permeability in human brain vascular cells (Hao et al., 2019). No data exists in an experimental neonatal HIE model but DPP-4 enzyme activity is known to increase in the blood serum of term and preterm neonates with cerebral ischemia (Yakovleva et al., 2015). Because DPP-4 inhibitors have shown neuroprotective properties and increase levels of GLP-1 in the brain, this could suggest that they have potential for treating HIE.

## Summary

The neuroprotective properties of diabetes drugs were first recognized by improvements in the neuropathic aspects in T2DM patients under treatment (Grant et al., 2011). The role of insulin as a pro-survival neurotrophic factor, where its receptor is widely expressed in cognitive areas of the brain such as the hippocampus and in the dopaminergic system also helped to consolidate this hypothesis (Haas et al., 2016; Fiory et al., 2019). The emerging evidence has suggested a beneficial effect of diabetes drugs in the management of diabetic and non-diabetic NDs. Furthermore, data supporting their neuroprotective effects are supported by a growing number of preclinical studies in neurodegenerative disorders such as AD (Cai et al., 2018; Dong et al., 2019) and PD (Athauda and Foltynie, 2016; Ayoub et al., 2018). Importantly, therapeutic efficacy had also been demonstrated in a clinical trial in PD patients (Athauda et al., 2017). However, many developmental, functional, and injury time course differences exist between the neonatal and the adult brain (Ferriero, 2004). Drug delivery properties, dosage and use can also be complex to translate from a adult to neonatal setting.

Several proof-of-concept studies with different classes of diabetes drugs as a treatment in neonatal HIE have been identified with glibenclamide (Zhou et al., 2009), metformin (Dadwal et al., 2015; Fang et al., 2017; Qi et al., 2017) and exendin-4 (Rocha-Ferreira et al., 2018). These diabetes drugs act on a plethora of biological pathways (Rosa et al., 2008; Wang M.-D. et al., 2012; Zhang et al., 2016; Zhu et al., 2016) and the precise mechanisms of action of diabetes drugs for neuroprotection are still not fully understood. However, in the context of HIE several studies have demonstrated neuroprotective actions of the GLP1-R agonists (Kimura et al., 2009; Jiang et al., 2016; Zhao et al., 2018) and metformin (Khallaghi et al., 2016) through the PI3K/Akt signaling pathway. In cases of brain injury, diabetes drugs have shown to be able to help repair the brain by modulating cell death mechanisms (Mielke et al., 2006; Gabryel and Liber, 2018; Xia et al., 2018), reducing neuronal oxidative stress (Mohammad Alizadeh et al., 2018) and promoting growth of new neurons and cells (Dadwal et al., 2015; Qi et al., 2017).

In adults, diabetes drugs are generally well tolerated with a long track record in the clinic and demonstrable safety profiles. However, this requires to be established in new born babies. Therefore, the normal advantages of rapidly repurposing drugs at a lower cost than the drug development process (Ayoub et al., 2018; Mousa and Ayoub, 2019) may be diminished for application to HIE. Potential safety risks could be reduced since the administration of the drug would likely only be required during a short acute period following the HI insult over a 48 h period (Rocha-Ferreira et al., 2018). However, depending on the diabetes drug in question, an important consideration is the potential to induce a hypoglycaemic effects when perturbations in glucose metabolism (hypoglycaemia and hyperglycemia) are already common in newborn infants with HIE (Vannucci, 1992; Salhab et al., 2004; Basu et al., 2009). This issue of hypoglycaemia exacerbating brain injury has been highlighted in the previously mentioned pre-clinical study using glibenclamide (Zhou et al., 2009). Therefore, the evaluation of the safety of diabetes drugs must be conducted in preclinical neonatal models, including larger animals prior to clinical trials.

## Conclusion

In conclusion, there is a growing body of evidence supporting the neuroprotective and anti-neuroinflammatory properties of specific diabetes drugs. Furthermore, the emerging proof of concept studies supporting their potential use as a treatment for HIE, either independently or in combination with hypothermia, is highly encouraging and warrants further investigation.

## Author Contributions

LP-B, ER-F, CT, HH, and AR drafted the manuscript.

## Conflict of Interest

The authors declare that the research was conducted in the absence of any commercial or financial relationships that could be construed as a potential conflict of interest.

## Abbreviations

AD: Alzheimer’s disease
BBB: blood brain barrier
DPP-4: dipeptidyl peptidase-4
DMB: quinoxaline 6,7-dichloro-2-methylsulfonyl-3-N-tert-butylaminoquinoxaline
GLP1: glucagon-like peptide-1
GLP1-R: glucagon like peptide-1 receptor
HI: hypoxia-ischemia
HIE: hypoxic-ischemic encephalopathy
HBMVECs: primary human brain microvascular endothelial cells
MAPK: mitogen-activated protein kinase
MCAO: middle cerebral artery occlusion
ND: neurological disorder
NPCs: neural precursor cells
OGD: oxygen glucose deprivation
OGD/R: oxygen glucose deprivation reperfusion
OPCs: oligodendrocyte progenitor cells
PD: Parkinson’s disease
PPAR- γ: peroxisome proliferator activated receptor- γ
ROS: reactive oxygen species
SUR: sulfonylurea
T2DM: type 2 diabetes mellitus
TH: therapeutic hypothermia
TBI: traumatic brain injury
TZD: thiazolidinedione
